# Varicose Veins of the Leg

**Published:** 1882-04-20

**Authors:** B. F. Duke

**Affiliations:** Mississippi


### VARICOSE VEINS OF THE LEG.

BY B. F. DUKE, M. D., OF MISSISSIPPI.

Dave G——, colored farm laborer, æt. 35, had his hand and arm severely torn by a gin-saw last November, from which he was forced to lie in bed about five weeks. During this time he had the good fortune to recover from a most distressing case of varicose veins of the leg, which had, for some time resisted treatment.

I had ordered a rubber bandage, intending to give it trial, but did not receive it until after the wound was inflicted by the gin. It was then kept firmly applied from toe to knee. Though I am satisfied that the cure was due mainly to the decubitus forced on the patient; and that had he been able to have pursued his daily work on the farm, the bandage, though a valuable auxiliary, would have been a failure, and the case continued one of those hopeless ones so often met with.

I have delayed reporting this case, which was one of great interest to me, expecting a recurrence, but up to this time there is no sign of it, though the man has been at hard work for two months.

